# Endoplasmic reticulum and Golgi stress in microcephaly

**DOI:** 10.15698/cst2019.12.206

**Published:** 2019-10-30

**Authors:** Sandrine Passemard, Franck Perez, Pierre Gressens, Vincent El Ghouzzi

**Affiliations:** 1 Université de Paris, NeuroDiderot, Inserm, F-75019 Paris, France.; 2 Service de Génétique Clinique, AP-HP, Hôpital Robert Debré, F-75019 Paris, France.; 3 Institut Curie, PSL Research University, CNRS, UMR144, Paris, France; 4 Centre for the Developing Brain, Division of Imaging Sciences and Biomedical Engineering, King's College London, King's Health Partners, St. Thomas'Hospital, London, United Kingdom.

**Keywords:** Golgi apparatus, endoplasmic reticulum, stress, UPR, corticogenesis, primary microcephaly, golgipathies

## Abstract

Microcephaly is a neurodevelopmental condition characterized by a small brain size associated with intellectual deficiency in most cases and is one of the most frequent clinical sign encountered in neurodevelopmental disorders. It can result from a wide range of environmental insults occurring during pregnancy or postnatally, as well as from various genetic causes and represents a highly heterogeneous condition. However, several lines of evidence highlight a compromised mode of division of the cortical precursor cells during neurogenesis, affecting neural commitment or survival as one of the common mechanisms leading to a limited production of neurons and associated with the most severe forms of congenital microcephaly. In this context, the emergence of the endoplasmic reticulum (ER) and the Golgi apparatus as key guardians of cellular homeostasis, especially through the regulation of proteostasis, has raised the hypothesis that pathological ER and/or Golgi stress could contribute significantly to cortical impairments eliciting microcephaly. In this review, we discuss recent findings implicating ER and Golgi stress responses in early brain development and provide an overview of microcephaly-associated genes involved in these pathways.

## INTRODUCTION

Brain size and how it has been tuned during evolution is a fascinating issue and studying cortical development in mammals represents a relevant approach to decipher the complex regulation of neural stem cells division and the pathological phenotypes associated with its failure, such as microcephaly. Microcephaly affects around 2% of the population worldwide and is one of the most frequent neurological signs encountered in neurodevelopmental disorders. It is characterized by a small brain size and is frequently associated with intellectual disability (ID) of variable severity. The dramatic increase in congenital microcephaly associated with the recent outbreak of Zika virus in Brazil as well as the identification of a large number of rare genetic forms of microcephaly over the last two decades have largely contributed to the understanding of the underlying mechanisms. They often involve a compromised division of cortical precursor cells affecting their neural commitment or survival and ultimately leading to a limited production of neurons. That brain size is determined by a fine regulation of cell number and the role of apoptosis has been exemplified in several mouse models: For example, mice haploinsufficient for *Magoh* display microcephaly due to an increase of neuronal apoptosis and a depletion of the intermediate progenitors [1]. Conversely, a decreased apoptosis results in overdevelopment of proliferative zones of the telencephalon and a markedly enlarged cerebrum in caspase 9 knockout (KO) mice [2] supporting the hypothesis that a precise balance between progenitor proliferation and apoptosis is required for normal corticogenesis. Ensuring neuronal homeostasis is essential and in this context the stress response has emerged as a key mechanism to adapt cells to the variations in cellular needs. Importantly, a large set of evidence indicates that most intracellular organelles are capable of auto-regulating their shape and functions and activate signaling pathways to adapt to cell status [3]. However, when the stress is abnormally prolonged or cannot be addressed by the organelle machinery (due to for example a genetic deficiency in a critical pathway), homeostasis and, with it, cell fate or viability may be rapidly compromised. In this review, we discuss recent findings implicating endoplasmic reticulum (ER) and Golgi apparatus (GA) stress responses in early brain development. We describe the signaling pathways that characterize the ER stress response and the more recently discovered Golgi stress response and provide an overview of microcephaly-associated genes involved in these pathways.

## CORTICOGENESIS IN MAMMALS

Corticogenesis is a complex and organized process regulated in time that takes place during pregnancy and results in the formation of the cortex from the dorsal neuroepithelium of the telencephalon. In humans, the cortex is thought to comprise more than 20 billion neurons [4], most of which are produced during the first two trimesters of pregnancy and migrate sequentially to create its laminar structure. At the very onset of corticogenesis, a single layer of neuroepithelial cells actively divides at the ventricular surface through symmetrical proliferative divisions aimed at amplifying the progenitor pool. These apical progenitors display apical-basal polarity and lie in between the ventricle and the basal lamina. As corticogenesis proceeds, neuroepithelial cells give rise to apical radial glial cells (aRGCs) that extend a process at each pole and further expand symmetrically but a number of which undergo asymmetric divisions that generate one self-renewed aRGC and one daughter cell committed to differentiation that delaminates from the apical surface and migrates basally [5]. This daughter cell may directly be a differentiating neuron (direct neurogenesis), a basal radial glial cell (bRGC) or an intermediate progenitor (IP) that will subsequently divide either asymmetrically to generate a neuron and a bRGC/IP or symmetrically to produce two neurons (indirect neurogenesis) [6, 7]. Controlling the balance between proliferative and differentiative divisions but also between direct and indirect neurogenesis is therefore critical to generate the proper number of neurons and achieve expansion of the cerebral cortex. While neuroepithelial cells and aRGCs divide perpendicularly to the ventricular surface of the neuroepithelium during proliferative divisions, the switch from symmetric to asymmetric divisions results from a deviation of the cell-division plane [8–10]. Oriented cell division is achieved through the proper positioning of the mitotic spindle, which largely depends on the microtubules emanating from the centrosomes at the two spindle poles and on pushing/pulling forces that are generated at the cell cortex [11, 12]. Further control of the balance between direct and indirect neurogenesis is also essential to corticogenesis. Direct neurogenesis largely predominates at early developmental stages, giving rise to neurons of the pre-plate and of deep cortical layers and then gradually decreases as corticogenesis proceeds, while indirect neurogenesis takes over and gives rise to younger neurons meant to upper cortical layers [13]. The selective depletion of IPs during corticogenesis results in marked microcephaly in mice indicating that indirect neurogenesis significantly contributes to cortical expansion, in addition to direct neurogenesis [14]. Thus, corticogenesis is a succession of tightly regulated proliferative steps and cell fate switches that creates different kinds of progenitors and generates distinct waves of projection neurons progressively contributing to the laminar layering of the cerebral cortex as well as to its radial and tangential expansion.

## PRIMARY MICROCEPHALY (PM)

Impairment of corticogenesis ultimately leading to a decreased production of neurons results in a congenital failure of brain growth called primary microcephaly (PM), a condition often associated with ID of variable severity. In clinics, PM is initially diagnosed through the measurement of the occipito-frontal circumference (OFC) of the head that increases during infancy following established curves. An OFC smaller than the age- and gender-adjusted mean by more than two standard deviations (SD) at birth is by definition indicative of PM. Underlying causes may be environmental factors (such as viruses, toxins such as alcohol, anoxia-ischemia or radiations) or genetic mutations [15, 16]. In particular, hereditary PM is frequent in neurodevelopmental disorders and highly heterogeneous: frequent, with more than 300 entries flagged by “Primary Microcephaly” in a search of Online Mendelian Inheritance in Man (OMIM); heterogeneous, with many distinct clinical pictures including variable severity of both brain size and intellectual abilities, presence or absence of epileptic seizures, occurrence of migration defects and/or of extra-cerebral signs such as skeletal growth retardation. Current classification of PM distinguishes conditions where microcephaly is the most prominent sign (MCPH - MicroCephaly Primary Hereditary) from those associated with primordial dwarfism such as Seckel or Meier-Gorlin syndromes or microcephalic osteodysplastic primordial dwarfism [17].

Despite this heterogeneity, several lines of evidence accumulated over the last years converge collectively toward mechanisms compromising chromosomal segregation and mitotic division especially in the developing cortex thereby leading to the exhaustion of the neural progenitor pool either through increased apoptosis or by premature differentiation and that results in PM. The various genes that have been associated with PM over the last 15 years have actually contributed greatly to the understanding of the main pathways involved in normal development and whose deregulation elicits PM [18]. Among them, the most studied involve genome integrity and cell cycle regulation.

## KNOWN MECHANISMS UNDERLYING PRIMARY MICROCEPHALY

### Genome instability and DNA damage

Mutations in several genes encoding DNA replication factors or DNA repair proteins have been identified in PM, pointing out genome instability as a frequent cause of PM [19–21]. DNA damage regularly and randomly arises in cells as an inevitable consequence of normal cellular processes. As an example, the spontaneous rate of production of endogenous DNA double-strand breaks (DSBs) in human cells has been estimated as high as 50 per cell and per cycle [22]. In most cases, this has no consequence on the cell survival, integrity and physiology as these DSBs are precisely and rapidly repaired by the DNA damage response (DDR), a process that comprises two major repair pathways named the homologous recombination pathway and the non-homologous end-joining (NHEJ) pathway [23], each of which include factors whose deficiency has been associated with PM [24]. In line with this, several mouse models lacking DNA replication factors or DNA end-joining proteins show embryonic lethality with microcephaly as a result of massive progenitor apoptosis, indicating that failure to repair leads to activation of programmed cell death pathways [25–28]. Interestingly, DDR activation acts through a temporary cell cycle arrest in G1/S or G2/M presumably to allow completion of DNA repair [23] and thus, DSBs repair appears strongly dependent on the cell-cycle state [29]. Disruption of the cell cycle control likely encourages the activation of programmed cell death pathways by interfering with DNA repair processes. However, the deregulation of cell cycle may result in PM by alternative mechanisms that do not necessarily imply cell death but instead result in premature neuronal differentiation.

### Cell cycle deregulation

Deregulation of the cell cycle control as a mechanism of PM is unambiguously illustrated by the *MCPH* genes, a family of 25 genes identified so far, most of which encode centrosomal, microtubule- or kinetochore-associated proteins; *MCPH* genes are highly expressed during brain development and in most cases their protein products are involved in the regulation of the mitotic spindle (including its formation and orientation), in the control of cell cycle or in the segregation of chromosomes especially during the division of progenitors of the ventricular zone at the onset of corticogenesis **(Table 1)**. Deficiency in *MCPH* genes causes PM and the most frequent form of PM, MCPH5, is associated with loss of function of ASPM (Abnormal spindle-like microcephaly-associated protein), a protein localized at the minus end of microtubules at the centrosome [30]. Defective ASPM has been associated with an increased frequency of asymmetric divisions at the expanse of symmetric divisions, resulting in a substantial reduction of the progenitor pool [31, 32]. Likewise, other MCPH-associated proteins such as WDR62 (MCPH2), CDK5RAP2 (MCPH3), CENPJ/CPAP (MCPH6), or STIL (MCPH7) play a role in centriole duplication, centrosome integrity and/or microtubule stabilization thereby regulating spindle positioning during mitosis [33–39]. Depending on the deficient MCPH protein, a variety of centrosomal defects can occur such as centriole overduplication, centriole disengagement or pericentriolar material fragmentation, often resulting in mitotic spindle multipolarity, which compromises chromosomal segregation in dividing cells and gives rise to aneuploidy [40]. In highly dividing progenitors these impairments in spindle integrity may have three deleterious consequences: (i) cell cycle arrest (ii) defects in chromosome segregation during mitosis and (iii) imbalance between symmetric and asymmetric divisions. While a prolonged cell cycle arrest will likely trigger apoptosis [41], the other two processes can lead to either progenitor cell death or their premature neuronal differentiation. Defects in chromosome segregation is known to lead to aneuploidy, the gain or loss of chromosomes, a situation associated with not only a variety of cancers but also microcephaly [42]. Aneuploid cells usually undergo apoptotic cell death [43, 44] and aneuploidy caused by overexpression of the centriole duplication protein Polo-like kinase 4 (PLK4) in mice results in microcephaly associated with premature cell death of the neural progenitors [45]. However, aneuploidy caused by the overexpression of the PLK4 homolog in Drosophila may also result in extended G1 phase, cell cycle exit and premature differentiation of neural stem cells [46]. Likewise, imbalance between symmetric and asymmetric divisions caused by the loss *in vivo* of several MCPH proteins such as ASPM, WDR62 or CDK5RAP2 results in premature neuronal differentiation of the progenitors in the developing neocortex [31, 35, 47], although apoptosis can also occur in certain circumstances [48].

**TABLE 1.** MCPH genes and proteins and their involvement in the regulation of the mitotic spindle, cell cycle control and/or chromosome segregation.

**Locus/***Gene name*	**Clinical features in humans**		**Protein**	**Intracellular Localization**	**Known Function**
	Major neurological signs	Short stature			
**MCPH1** *MCPH1*	Severe PM, ID, premature chromosome condensation	++	MICROCEPHALIN	Nucleus, cytoplasm, centrosome depending on the isoform and cell type	DNA Damage response, transcription, cell cycle control, Spindle pole orientation
**MCPH2/***WDR62*	Normal OFC to severe PM, seizures, spastic quadriparesis, severe ID, cortical malformations (polymicrogyria, schizencephaly, nodular or subcortical heterotopia)	no	WDR62	Spindle pole, centrosome, centriole, nucleus	Centriole duplication, spindle pole orientation
**MCPH3/***CDK5RAP2*	Severe PM, sensorineural hearing loss, ID	+	CDK5RAP2	Centrosome (PCM)	Spindle pole orientation, PCM maturation, microtubule nucleation, Centriole engagement
**MCPH4/***KNL1*	Severe PM, ID	no	KNL1	Kinetochore	Kinetochore, microtubule attachment
**MCPH5/***ASPM*	Severe PM, ID, gyral simplification	no	ASPM	Spindle pole	Spindle pole orientation & integrity
**MCPH6/***CENPJ*	Severe PM, short stature, Seckel syndrome, ID	+++	CENPJ	Centriole	Centriole duplication
**MCPH7/***STIL*	Severe PM, ID	+++	STIL	Centriole	Centriole duplication
**MCPH8/***CEP135*	Severe PM, ID	++	CEP135	Centriole	Centriole duplication
**MCPH9/***CEP152*	Severe PM, ID	+++	CEP152	Centriole	Centriole duplication
**MCPH10/** ZNF335	Severe PM, ID, seizures, brainstem hypoplasia	no	ZNF335	Nucleus (histone methyltransferase complex protein)	Transcription
**MCPH11/***PHC1*	Severe PM, ID	+	PHC1	Nucleus (Polycomb group multiprotein PRC1-like complex protein)	Transcription
**MCPH12/***CDK6*	Severe PM, ID	no	CDK6	Nucleus (kinase activity), centrosome	Cell cycle control
**MCPH13/***CENPE*	Severe PM, ID, gyral simplification, cerebellar hypoplasia	+++	CENPE	Kinetochore, mitotic spindle	Kinetochore microtubule attachment, chromosomes congression
**MCPH14/***SASS6*	Severe PM, ID	++	SASS6	Centriole	Centriole duplication
**MCPH15/***MFSD2A*	Severe PM, seizures, spastic tetraparesis, hydrocephaly, thin cortex, brainstem hypoplasia	no	MFSD2A	BBB in endothelial cells	Brain uptake of DHA / fatty acids
**MCPH16/***ANKLE2*	Severe PM, ID, seizures, spastic tetraparesis	++	ANKLE2	Nuclear envelope, ER	Nuclear envelope reassembly in late anaphase
**MCPH17/***CIT*	Severe PM, ID, spastic tetraparesis, microlissencepaly	no	CIT	Midbody	Cytokinesis
**MCPH18/***WDFY3*	PM, ID	na	WDFY3	Autophagic structures	Macroautophagy
**MCPH19/***COPB2*	Severe PM, ID, spastic tetraparesis, cortical blindness, gyral simplification	no	COPB2	Golgi coatomer complex COPI	Retrograde Golgi to ER transport of vesicles
**MCPH20/***KIF14*	Severe PM, ID, spastic tetraparesis, gyral simplification	+	KIF14	Microtubule motor protein, microtubules, spindle pole, midbody	Cytokinesis Chromosome congression,
**MCPH21/***NCAPD2*	Severe PM, severe ID, seizures, autism	+++	NCAPD2	Chromatin (condensin multiprotein complex)	Chromatin condensation during mitosis
**MCPH22/***NCAPD3*	Severe PM, mild to severe ID, seizures	++	NCAPD3	Chromatin (condensin multiprotein complex)	Chromatin condensation during mitosis
**MCPH23/***NCAPH*	PM, ID	no	NCAPH	Chromatin (condensin multiprotein complex)	Chromatin condensation during mitosis
**MCPH24/***NUP37*	Severe PM, mild ID, vermis hypoplasia	no	NUP37	Nuclear envelope (NPC), kinetochore during mitosis	NPC, kinetochore microtubule attachment
**MCPH25/***MAP11*	Severe PM, ID	no	MAP11	Microtubule associated protein, spindle pole	Spindle dynamics

MCPH proteins for which no association with the mitotic spindle, cell cycle control and/or chromosome segregation has been documented so far are mentioned in light grey background. PM: Primary Microcephaly; ID: intellectual disability; OFC: occipito-frontal circumference; PCM: pericentriolar matrix; BBB: blood-brain barrier, DHA: docosahexaenoic acid; ER: endoplasmic reticulum; NPC: nuclear pore complexes; na: non-available.

## ER STRESS IN PRIMARY MICROCEPHALY

### Pathways associated with ER stress

**Figure 1:**
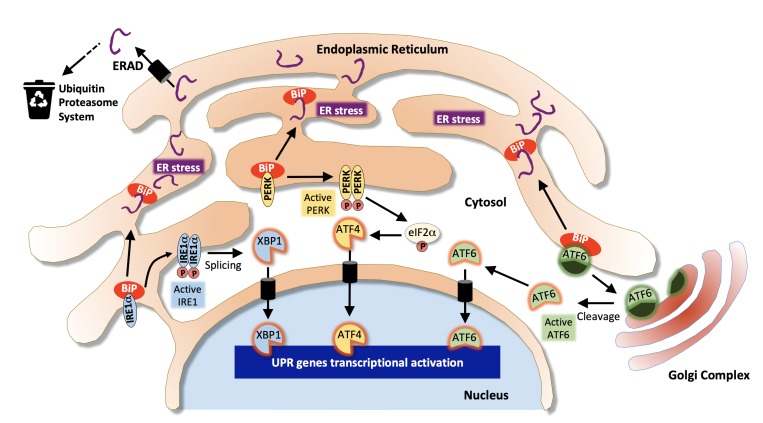
FIGURE 1: Pathways associated with the Endoplasmic Reticulum stress response. Misfolded proteins are targeted to the ER-associated degradation machinery (ERAD) that sends them to the ubiquitin proteasome system for cytosolic degradation. Excessive accumulation of unfolded/misfolded proteins results in ER stress and dissociation of BiP from IRE1α, PERK and ATF6 leading to their activation and that of the unfolded protein response (UPR). The IRE1α/XBP1 pathway (in blue) leads to XBP1-dependent activation of chaperones and folding enzymes; The PERK/ATF4 pathway (in yellow) favors the activation of pro-apoptotic genes such as CHOP. Activation of eiF2α also contributes to translation inhibition. In the ATF6 pathway (in green), ATF6 is first transported to the Golgi where it is activated by proteolytic cleavage and translocates to the nucleus to activate the expression of XBP1 and other target genes involved in ERAD. (Adapted from [139]).

As the main site of protein synthesis, the ER permanently faces important flows of nascent polypeptides that need to be properly folded, matured and transported. These processes are helped by a number of chaperone proteins (such as heat shock proteins from the Hsp70 and Hsp90 families or the glucose-regulated protein BiP/GRP78) and folding enzymes (such as oxidoreductases or the protein disulfide isomerase, PDI) that are critical in maintaining ER proteostasis [49, 50]. Misfolded proteins are guided to the ER-associated degradation (ERAD) machinery that retro-translocates them back to the cytosol where they are targeted to the ubiquitin proteasome system to be degraded [51]. Failure to degrade misfolded protein leads to their accumulation in the ER lumen and results in ER stress and the activation of the unfolded protein response (UPR), an adaptive transcriptional program aiming at restoring proteostasis by enlarging ER membranes, limiting ER protein loading by inhibiting protein translation and promoting chaperone expression and ERAD activation [52] **(Figure 1)**. However, when the UPR faces a significant overload of misfolded cargos and fails to normalize the situation it ultimately mediates apoptotic cell death [53]. In mammalian cells, the UPR is mediated by three ER-to-nucleus signaling pathways initiated by distinct ER membrane receptors, the inositol-requiring enzyme 1 alpha (IRE1α), the protein kinase R-like endoplasmic reticulum kinase (PERK also known as EIF2AK3) and the activating transcription factor 6 (ATF6) [54]. These transmembrane receptors act as sensors of ER stress and are kept inactive in basal condition through the interaction of their luminal domain with BiP/GRP78. Under ER stress, BiP/GRP78 dissociates from IRE1α, PERK and ATF6 leading to their activation [52]. Upon activation, IRE1α mediates the nuclear translocation of the XBP1 transcription factor, PERK phosphorylates the α subunit of eukaryotic initiation factor 2 (eIF2α) which promotes the translation of the ATF4 transcription factor and ATF6 is released from its membrane anchor and also translocates into the nucleus to activate gene expression **(Figure 1)**. In addition to its role in the PERK-eIF2α-ATF4 arm of the UPR, eIF2α lies at the core of the translation initiation machinery as one of the three subunits α, β and γ of the eukaryotic translation initiation factor 2 (eIF2) complex. eIF2 associates with GTP and the initiator methionyl-tRNA, and binds to the small 40s ribosomal subunit to participate in protein translation [55]. Phosphorylation of eIF2α is involved in this process as it inhibits eIF2B, the guanine nucleotide exchange factor of eIF2, thereby controlling the GTP-dependent activity of the complex. Thus, the regulation of protein translation initiation is linked to that of ER stress and to the PERK-eIF2α-ATF4 arm of the UPR.

### ER proteostasis during corticogenesis

Although ER proteostasis is important in all cells and tissues, its particular role during brain development, especially during corticogenesis, has been highlighted by several studies suggesting that ER stress and the UPR physiologically participate in embryonic cortical development. Several stress-regulated chaperones and folding enzymes including BiP/GRP78, GRP74, PDI and Calreticulin were found particularly abundant in mouse embryonic cerebral cortex as compared to adult brain, and all three UPR pathways are activated during CNS (central nervous system) development, including in the ventricular zone of the developing cortex [56–58]. In agreement with a key role in early brain development, embryos lacking Calreticulin display neural tube closure defects and die in late-gestation [59]. The complete deletion of BiP/GRP78 also results in embryonic lethality [60] and partial reduction of BiP function following hypomorphic mutations of the gene results in severe microcephaly due to abnormal corticogenesis [61, 62]. Mouse embryonic fibroblasts (MEFs) where the ER targeting of BiP is hampered by the lack of its KDEL retrieval motif showed an increased expression of XBP1, ATF4 and phospho-PERK, indicating that the UPR is constitutively activated [63]. These data raise the possibility that the dynamic regulation of ER stress and UPR could contribute to the control of corticogenesis. Using an elongator complex component (Elp3) conditional knockout mouse where the PERK-eIF2α-ATF4 signaling branch of the UPR is specifically upregulated in cortical progenitors, Laguesse and colleagues [64] discovered a role of the UPR in controlling the balance between direct and indirect neurogenesis. They showed that a prolonged UPR in cortical progenitors favors direct neurogenesis of apical progenitors (aRGCs) at the expanse of IPs, leading to an impairment of indirect neurogenesis and ultimately resulting in microcephaly in mice [64]. This suggests that gradual suppression of the UPR is physiologically required to allow indirect neurogenesis and that a dynamic regulation of UPR controls corticogenesis.

### Primary microcephaly associated with ER stress pathways deregulation

In human as well, several lines of evidence indicate that interfering with ER stress pathways during brain development results in neurodevelopmental disorders including PM. For example, alcohol dependence during pregnancy can induce fetal alcohol spectrum disorders (FASD), a condition commonly associated with PM and ID, due to high vulnerability of the immature brain to ethanol exposure [65]. A deregulation of ER stress and UPR including a prolonged activation of the PERK-eIF2α-ATF4 and ATF6 pathways has been observed in both *ex vivo* and *in vivo* models of ethanol-induced neuronal cell damage [66–68] that could contribute significantly to the neuronal loss observed following ethanol exposure [69, 70]. Alcohol has been proposed to induce ER stress through various mechanisms involving epigenetic alterations, homocysteinylation of proteins, generation of abnormal protein adducts or perturbations of calcium homeostasis [71]. ZIKA virus (ZIKV) also causes severe PM in newborns when mothers get infected during early pregnancy [72–74] and massive cell death and ER damage have been described in the subventricular zone and cortical plate from ZIKV-infected fetuses [75]. ZIKV triggers ER stress and UPR both in human cortices *in vivo* and in human neural stem cells *ex vivo* and contributes to the development of microcephaly in mice, mainly through over-activation the PERK-eIF2α-ATF4 pathway, which (as seen in the Elp3 knock out model) results in an impairment of indirect neurogenesis [76]. In line with these findings, genetic mutations in several components of the PERK-eIF2α-ATF4 pathway were shown to result in rare autosomal recessive syndromes that include congenital microcephaly among their symptoms: PERK/EIF2AK3 deficiency that impairs eIF2α phosphorylation causes Wolcott-Rallison syndrome (#MIM226980) characterized by early-onset insulin-dependent diabetes associated with growth retardation, hepatic dysfunction, pancreas insufficiency and microcephaly with ID [77]. Homozygous mutations in the eIF2α phosphatase gene *PPP1R15B* result in increased eIF2α phosphorylation and stress resistance and also cause microcephaly, short stature and impaired glucose metabolism (MSSGM2, #MIM616817) [78, 79]. Protein synthesis was apparently not affected by *PPP1R15B* mutations, at least *in vitro* [78]. Interestingly, while mutations in *ATF4* have not been reported in human pathology, forced expression of the gene in Xenopus embryos resulted in severe microcephaly with absence of eyes [80].

The contribution of eIF2α in both UPR and translation initiation suggests that both processes are regulated at least in part by common factors and that a deficiency in translational accuracy could also result in microcephaly in humans. Indeed, mutations in *EIF2S3*, the gene encoding eIF2γ that was shown to compromise eIF2 complex integrity and translation initiation, cause MEHMO (#MIM300148), a syndrome associating epilepsy, hypogonadism, obesity and microcephaly with ID [81]. Loss-of-function mutations in *OSGEP*, *TP53RK*, *TPRKN* or *LAGE3*, which encode the four subunits of the KEOPS complex (Kinase, Endopeptidase and Other Protein of small Size) result in Galloway-Mowat syndrome (GAMOS, #MIM251300), an early-onset nephrotic disorder with severe PM [82]. The KEOPS complex is required for a universal modification called threonyl carbamoyl adenosine found in all tRNAs in eukaryotes and necessary for translational efficiency [83]. CRISPR/Cas9 disruption of these genes recapitulated the microcephaly in mice and zebrafish and knockdown of *OSGEP*, *TP53RK*, *TPRKN* resulted in impaired protein translation, ER stress and the activation of the PERK-eIF2α-ATF4 and IRE1α-XBP1 pathways [82]. Thus, like in the Elp3 conditional knockout mouse published by Laguesse and colleagues [64], a defect in the translation process speed or accuracy in human may trigger ER stress and subsequent UPR and result in multiple organ defects including brain and PM.

## GOLGI STRESS IN MICROCEPHALY

### Pathways associated with Golgi Stress

The GA is a highly dynamic organelle that permanently adapts its morphology and processing capacities in response to secretory flows depending on cell demand. In several pathologies where its secretory function is overwhelmed or compromised, including neurodegenerative and neurodevelopmental diseases, the GA appears swollen and/or fragmented [84, 85]. Of course, owing to its role in post-translational modifications of newly synthesized proteins and lipids and because of its close relation with the ER in both secretory and retrograde routes, the GA is often impacted by a stress occurring initially in the ER. This is, for example, well-illustrated by loss-of-function mutations recently identified in ARCN1 and SEC31A, two subunits respectively constitutive of COPI and COPII, the Coat protein complexes that mediate retrograde and anterograde transport of the vesicles transiting between the ER and the GA and which, in addition to triggering ER stress and trafficking defects, have been associated with primary microcephaly [86, 87].

However, defects observed at the level of the GA do not always result from alteration of ER functions. It is now clear that the GA can directly initiate and modulate signalling cascades [88], and the increasing evidence of a close relationship between signaling and changes in Golgi size and architecture suggest a role for the GA as a cell sensor [89]. Fifteen years ago, Hicks and Machamer hypothesized a Golgi stress response capable of adapting the capacity of the GA to cellular demand [90]. Emerging evidence now support that in response to a stress that modifies its pH, impedes glycosylation, sialylation or other modifications, or interferes with vesicle transport, the GA adapts its morphology and, like the ER, is able to elicit independently the activation of specific transcriptional programs aimed at increasing its capacities and restoring homeostasis [91].

**Figure 2:**
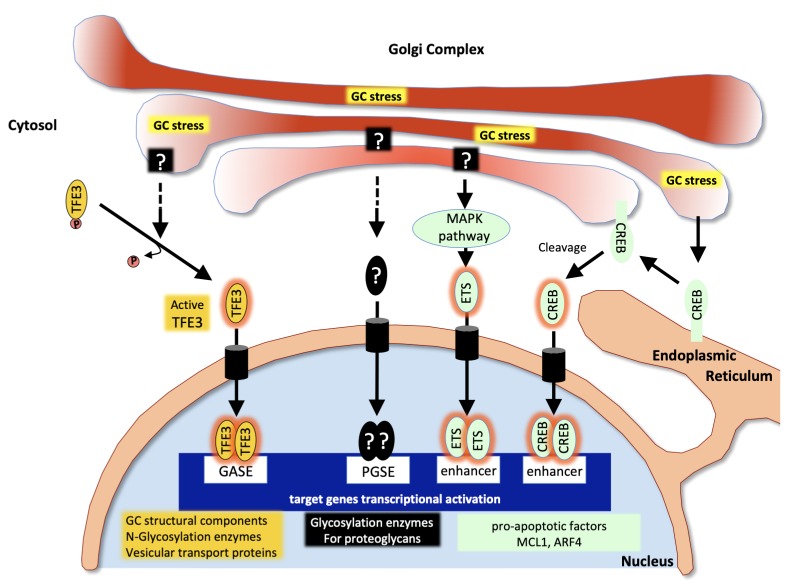
FIGURE 2: Pathways associated with the Golgi stress response. Upon Golgi stress, several specific transcriptional programs are activated. One pathway (in yellow) is mediated by the transcription factor TFE3 which translocates to the nucleus and activates genes encoding Golgi structural components, glycosylation enzymes and vesicular transport proteins all containing GASE in their promoter. The proteoglycan pathway (in black) is induced by insufficiency in the capacity to glycosylate proteoglycans. Sensors at the Golgi and factors mediating this pathway are still to be identified but contribute to the activation of genes encoding proteoglycan-specific glycosylation enzymes all containing a PGSE consensus sequence in their promoter. The MAPK/ETS pathway and the CREB3 (in green) both activate pro-apoptotic genes in response to Golgi stress. (Adapted from [140]).

A first Golgi stress pathway is mediated by the bHLH leucine zipper transcription factor E3 (TFE3). TFE3 expression increases upon Golgi stress such as monensin (MON) treatment (a drug that neutralizes the acidic pH of the GA thereby strongly reducing the activity of resident proteins) and the transcription factor translocates into the nucleus. There, it activates the expression of genes that include in their promoter the consensus sequence "ACGTGGC" called the Golgi apparatus stress response element (GASE) [92] **(Figure 2)**. These target genes encode several glycosylation enzymes (such as sialyltransferases or the fucosyltransferase FUT1), as well as factors involved in Golgi structure and vesicular transport such as WIPI49, ACBD3, the golgins GM130 and Giantin, the small GTPase Rab20 and Syntaxin 3A [93]. The pathway appears negatively regulated by MLX, another bHLH leucine zipper transcription factor that is also activated and translocated to the nucleus upon Golgi stress and can competitively bind to GASE and modulate the expression of the same target genes [94]. Although the upstream sensor molecules of the TFE3 pathway have not yet been identified, a recent study by Serebrenik and colleagues [95] has shown, by inducing protein destabilization in the Golgi without affecting the ER, that the GA is able to sense unfolded proteins just as the ER does. Protein unfolding in the Golgi resulted in the up-regulation of a set of Golgi-related genes several of which are induced by the TFE3 pathway. This suggests that in addition to known Golgi stress inducers affecting pH of the GA or its secretory functions, protein unfolding can elicit a Golgi-specific unfolded protein response and that this Golgi UPR likely includes the TFE3 pathway. In line with this, TFE3 controls the transcription of autophagy genes in response to cellular stress as in the case of pancreatic cancer or heavy metals intoxication [96, 97] and promotes autophagy, lysosomal biogenesis and clearance of cellular debris [98].

GA dispersal and stress are also known to occur in response to various Golgi-disrupting drugs such as brefeldin A (BFA) or golgicide A (GCA) in addition to MON, and the study of their mechanisms of action has considerably helped identifying key factors involved in the regulation of Golgi morphology and activation of stress response. A second signaling pathway mediated through the CREB3 transcription factor has indeed been uncovered thanks to a genetic screen for resistance to BFA-induced toxicity [99]: The ADP-ribosylation factor ARF4, a member of the Ras superfamily of small G proteins mediates the response to Golgi stress as its depletion protects the cells from undergoing apoptosis induced by BFA, MON or GCA [99]. In this study Reiling and colleagues showed that Golgi stress resulting from these drugs causes ARF4 induction mediated through CREB3. CREB3 belongs to the basic leucine zipper (bZIP) family of transcription factors and is activated upon proteolytic cleavage in the GA and translocated into the nucleus where it activates the transcription of its target genes, in particular during CNS development [100]. Gene expression profiling following treatment with the same Golgi disruptors and causing ARF4 up-regulation further identified a MAPK signaling pathway regulating the pro-apoptotic splicing of the apoptosis regulator MCL1 through the activation of the three ETS transcription factors ELK1, GABPA and ETS1 [101] **(Figure 2)**. Although this pathway has been identified in the context of a pharmacological disruption of the GA, it is possible that disease-associated Golgi fragmentation triggers physiological stress through the CREB3-ARF4 pathway, followed if the stress cannot be resolved, by MCL1-mediated apoptosis through this MAPK pathway.

Also identified in the same genetic screen for resistance to BFA-induced toxicity [99], TRAPPC13 appears to modulate the response to Golgi stress as well. TRAPPC13 is a subunit of the multimeric transport protein particle (TRAPP) complex involved in the regulation of ER-to-Golgi and intra-Golgi traffic [102, 103]. Knock down of TRAPPC13 protected cells from cell death caused by prolonged BFA, MON or GCA treatment and correlated with a reduced rate of autophagy, suggesting a role of TRAPPC13 in autophagy and a role of autophagy in cell death mediated by a prolonged Golgi stress [104]. Ramírez-Peinado and colleagues also found that the resistance of TRAPPC13-depleted cells to such Golgi stress was dependent on ARF1 activity, indicating a genetic interaction between TRAPPC13 and ARF1. Very interestingly, several other TRAPP complex subunits such as TRAPPC3, TRAPPC9, TRAPPC11 and TRAPPC12 behaved similarly to TRAPPC13 towards BFA- and GCA-induced toxicity, suggesting that several TRAPP complex components are involved in the Golgi stress response [104].

Alternative Golgi stress pathways could be initiated by the abnormal accumulation of certain compounds in the GA that are capable of activating a signaling cascade. Such a mechanism involving abnormally/insufficiently glycosylated proteoglycans has been proposed by Hiderou Yoshida [105]. Treatment with xyloside causes sequestration of the immature proteoglycans in the GA and subsequent swelling and fragmentation [106]. Instead of undergoing degradation, proteoglycans such as Syndecan2 may behave as Golgi stress inducers and trigger the activation of target genes, many of which encode proteoglycans glycosylation enzymes, under the control of what has been called a proteoglycan-type Golgi stress response elements (PGSE) **(Figure 2)**. Another pathway triggered by diminished levels of glycosylation enzymes for mucins has been reported very recently [107]. This mucin pathway stimulates the expression of a number of N-Acetylgalactosaminyltransferases important for mucin glycosylation through mucin-type Golgi stress response elements (MGSE) and appears to crosstalk signal to the TFE3 pathway. These mechanisms further illustrate the adaptive strategies undertaken by the GA to restore cell homeostasis.

Recent studies have revealed a role for several other Golgi proteins in sensing distress potentially caused by glucose starvation, oxygen deprivation or DNA damage, indicating that the GA is sensitive to a broad range of cell stressing agents and suggesting that additional signaling pathways activated in response to these situations remain to be identified. The Golgi reassembly stacking protein GRASP55, whose main known function in mammalian cells is to ensure the lateral linking of Golgi stacks into a single ribbon, is relocated to the autophagosome-lysosome interface upon glucose starvation where it promotes autophagosome maturation [108, 109]. GOLPH3, another membrane protein of the Golgi mainly localized in the *trans*-Golgi network (TGN), also plays a role in the ribbon structure and binds phosphatidylinositol-4-phosphate (PtdIns-4-P) and actomyosin to stretch the Golgi and promote budding [110], but relocates to cytoplasmic vesicle-like structures and promotes autophagy in response to stress caused by oxygen-glucose deprivation and prolonged reoxygenation [111]. GOLPH3 also promotes cell survival following DNA damage [112, 113] and behaves as an oncoprotein being overexpressed in many aggressive cancers and promoting cell survival and proliferation through several pathways including mTOR, Wnt/β-catenin and JAK/STAT pathways [114–117].

### GA function during corticogenesis

In addition to its canonical functions in processing newly synthesized proteins and lipids and regulating their sorting and routing to their final destination in most cells, the GA possess particular features specific to aRGCs that underpin a crucial role of the GA in maintaining neural stem cell polarity that may have consequences for neural progenitor cell fate transition during corticogenesis in mammals [118]. By contrast to basal progenitors (such as bRGCs or IP as mentioned above), aRGCs maintain contact with both the ventricle surface (apical pole) and the basal lamina (basal pole), which makes them unique bipolar epithelial cells. Unlike the ER that is detected in both apical and basal processes, the GA of aRGCs remains confined to their apical process, apical to the nucleus irrespective of its position along the apical-basal axis and irrespective of the cell-cycle phase. It is not pericentrosomal as it usually is in most interphase cells (including bRGCs and IP) and disassembles earlier and reassembles later in mitosis than the GA of basal progenitors [118]. Although the precise purpose of this specific segregation of the GA to the apical compartment remains to be understood, it has been proposed that this optimizes apical membrane trafficking or participates in specific apical signaling during corticogenesis [119]. In their study, Xie *et al.* showed that this apical distribution of the GA is mediated by a lipid signaling pathway, involving the two highly related lipid transfer proteins PITPNA and PITPNB. PITPNA/PITPNB potentiate the PtdIns-4-P-dependent recruitment of GOLPH3 to Golgi membranes, which in turn interacts with MYO18A and F-actin to direct the loading of the GA to apical compartments [119]. In a more recent study, Rahajeng *et al.* described how GOLPH3 induces Golgi membrane curvature upon binding to PtdIns-4-P-rich lipid bilayers and how this membrane-shaping function of GOLPH3 is a pre-requisite to enable efficient anterograde trafficking through its direct interaction with MYO18A [120]. Collectively, these studies confer a particular importance of the GA in the maintenance of neural stem cell identity throughout the development of the neocortex. The central role played by GOLPH3 upon energetic stress, its implication in cell survival following DNA damage and its involvement in the apical identity of aRGCs during neocortical development, suggest that GOLPH3-controlled pathways may be strongly associated with microcephaly. Interestingly, GOLPH3 appears also to be required for proper cell cycle progression as its depletion with small interfering RNA delays the G1 to S transition in U2OS cells [121]. Furthermore, simultaneous loss of PITPNA and PITPNB in the mouse neocortex results in the absence of the dorsal forebrain due to marked alignment defects of radial glial progenitors that coincides with the loss of the apical distribution of the GA and precedes massive apoptosis in double KO aRGCs [119].

### Microcephaly and Golgi stress pathway deregulation

Among the growing number of Golgi-related genes known to be associated with disease (Golgipathies) over 40% affect the central or peripheral nervous systems, highlighting the critical importance of the GA to neural function [122, 123]. As expected from the critical functions driven by the GA during brain development and maturation, neurodevelopmental Golgipathies frequently include microcephaly and ID among their symptoms. While the first identified microcephalies primarily associated with Golgi deficiency were postnatal [85], a number of Golgipathies causing early onset PM have since then been identified [123]. Pathways associated with Golgi stress have emerged only recently and as they are just starting to be elucidated at the molecular level, the potential connection between PM and Golgi stress is only beginning to be unraveled.

For example, following TFE3 translocation into the nucleus various target genes are activated, among which are a number of glycosylation enzymes such as sialyltransferases and fucosyltransferases as mentioned above. Interestingly, abnormal fucosylation and sialic acid deficiency have been reported in association with congenital disorder of glycosylation type IA (CDG-Ia; MIM #212065), the most common form of CDG (Congenital Disorders of Glycosylation) [124, 125]. CDG-Ia is a multisystem disorder that has been associated with progressive microcephaly [126]. Interestingly, loss of the golgin GM130, a multifunctional golgin involved in the maintenance of Golgi structure and the regulation of the secretory pathway and whose expression is also targeted by TFE3, leads to a neuromuscular syndrome with microcephaly in humans, a phenotype recapitulated in zebrafish [127]. In agreement with these observations, knocking out GM130 in mice results in Golgi fragmentation and neuronal loss [128]. However whether Golgi stress pathways are activated and deregulated in these models has not yet been investigated.

Another example has been reported by Sheen and colleagues who identified loss-of-functions mutations in the ADP-ribosylation Factor Guanine Exchange Factor 2 (*ARFGEF2*) in patients with an autosomal recessive periventricular heterotopia with microcephaly (ARPHM; MIM #608097) [129]. *ARFGEF2* encodes BIG2, a guanine nucleotide-exchange factor involved in vesicle trafficking between endosomes and the TGN during cortical development [130]. A more recent study performed in hippocampal neurons suggests that BIG2 regulates dendritic Golgi polarization and controls dendritic growth and maintenance through ARF1 [131]. BIG2 also directly binds to ARF4, which mediates its recruitment at TGN membranes [132]. Loss of function of BIG2 impairs the proliferation of neural progenitors as shown by BFA treatment or expression of dominant negative BIG2 mutants [129]. Treating cells with GCA also results in BIG2 loss of function as it inhibits its recruitment at TGN membranes, a situation that is restored upon overexpression of the active form of ARF4 [133]. Thus, it is possible that BIG2 participates in the ARF4-dependent Golgi stress signaling cascade, linking microcephaly to Golgi stress. In line with this, inhibition of CREB in Xenopus or Zebrafish embryos results in decrease of neural cell proliferation and microcephaly [134, 135].

The TRAPP complex, which in addition to its role as tether also modulates the response to Golgi stress through several of its subunits as mentioned above, was first identified in yeast where three related complexes have been described. Despite a high degree of conservation with yeast, only two complexes, homologous of TRAPP II and III have been reported so far in mammals [103]. In human, TRAPP II and III share common TRAPP subunits (TRAPPC1 to 6) and differ by TRAPPC9 and 10 (specific to TRAPP II) and TRAPPC8, 11, 12 and 13 (specific to TRAP III). Interestingly, mutations in both core and specific TRAPP subunits have been identified in autosomal recessive developmental disorders that include progressive microcephaly [123]. TRAPPC2L, a close variant of TRAPPC2 has been recently involved in a developmental delay with postnatal microcephaly, dystonia, tetraplegia, rhabdomyolysis, encephalopathy and epilepsy [136]. Similarly, the core protein TRAPP6B is deficient in a neurodevelopmental disorder with postnatal microcephaly, epilepsy and brain atrophy (NEDMEBA, MIM #617862)[137]. Although the link between the response to Golgi stress and genetic mutations in these core TRAPP proteins remains to be investigated, mutations in TRAPPC9, TRAPPC11 and TRAPPC12, which are specific to either the TRAPP II complex (TRAPPC9) or the TRAPP III complex (TRAPPC11 and 12) and mediate BFA- and GCA-induced Golgi stress, have been reported in microcephalic patients [123]. Loss of function mutations in TRAPPC9 result in an autosomal recessive mental retardation (MRT13, MIM #613192) including a moderate-to-severe postnatal microcephaly, a peculiar facial appearance, obesity and hypotonia with associated cerebral white matter defects. Mutations in TRAPCC11 lead to limb girdle muscular dystrophy (LGMDR18, MIM #615536) with microcephaly of unknown onset, intellectual deficiency, myopathy and ataxia. Mutations in TRAPPC12 result in progressive childhood encephalopathy (PEBAS, MIM #617669) with microcephaly and white matter defects and severe developmental delays [138]. The high heterogeneity in the developmental aspects involved in these various TRAPP-associated disorders highlights the multiple roles that TRAPP proteins likely play in vesicle tethering, autophagy and in various aspects of Golgi dynamics in different organs. The recurrence of microcephaly in these disorders as well as its progressive nature raise the question of whether the global and persistent perturbation of Golgi homeostasis could be a hallmark of microcephaly.

## CONCLUSION

Pathways associated with ER stress are by far more studied and have received more attention than those associated with Golgi stress which are only starting to be deciphered. It has become obvious nevertheless that both organelles dynamically and specifically react to various cell stresses to maintain proper cellular homeostasis. Why brain development would be so strongly affected by defects in ER/Golgi stress control is still an open question. One key particularity of neocortical development is that neural stem cells need to divide and differentiate actively in a short period of time and according to a spatially regulated pattern, which involves sequential waves of progenitors and neurons production. Hence, a deregulation of ER or Golgi stress pathways in the developing brain may understandably end in catastrophic outcome. In addition, brain development strongly relies on highly polarized cells at every step of corticogenesis from the first neuroepithelial cells to aRGCs, bRGCs and to neurons. As polarity establishment and maintenance are both highly dependent on traffic through the secretory pathway, an impairment of cell polarity could be one of the reasons to explain the particular vulnerability of the brain to ER/Golgi stress. Proper control of the extracellular environment, stem cell niches, may also be particularly important and rely on intense and proper function of the secretory pathway in such a short time. This pinpoints the essential adaptive capacities of the mammalian brain in addressing and solving the permanent aggressions and disequilibrium that it experiences and suggests that additional cell homeostasis pathways, whose deregulation might be associated with microcephaly, will be identified in the near future.

## Abbreviatons:

aRGC: – apical radial glial cell,
BFA: – brefeldin A,
bRGC: – basal radial glial cell,
CDG: – Congenital Disorders of Glycosylation,
CNS: – central nervous system,
DDR: – DNA damage repair,
DSB: – double-strand break,
ER: – endoplasmic reticulum,
ERAD: – ER-associated degradation,
GA: – Golgi apparatus,
GASE: – Golgi apparatus stress response element,
GCA: – golgicide A,
ID: – intellectual disability,
IP: – intermediate progenitor,
KEOPS: – Kinase, Endopeptidase and Other Protein of small Size,
MON: – monensin,
OFC: – occipito-frontal circumference,
PM: – primary microcephaly,
TGN: – trans-Golgi network,
TRAPP: – transport protein particle,
UPR: – unfolded protein response,
ZIKV: – ZIKA virus.
